# Novel PASC-based genotyping of goose astrovirus using ORF2 amino acid sequences

**DOI:** 10.3389/fvets.2026.1732594

**Published:** 2026-10-27

**Authors:** Zhibin Zhang, Haoyang Wang, Xin Xu, Dandan Li, Jun Ji, Lunguang Yao, Yingzuo Bi, Qingmei Xie

**Affiliations:** 1 Henan Provincial Engineering Laboratory of Insects Bio-reactor, Henan Provincial Engineering, and Technology Center of Health Products for Livestock and Poultry, Henan Provincial Engineering and Technology Center of Animal Disease Diagnosis and Integrated Control, Nanyang Normal University, Nanyang, China; 2 College of Animal Science, South China Agricultural University, Guangzhou, China

**Keywords:** genotype analysis, goose astrovirus, ORF2, PASC genotyping, subtype classification

## 1 Introduction

Astroviruses are non-enveloped viruses with single-stranded positive-sense RNA (+ssRNA, 6–7 kb in length) genomes, icosahedral symmetry and a capsid approximately 30 nm in diameter (1). Astroviruses infect a broad range of hosts, with members of the genus Mamastrovirus infecting mammals and members of the genus Avastrovirus infecting birds (2). Novel Goose astrovirus (N-GoAstV) belongs to the Avastrovirus genus. Since its first report in 2017, N-GoAstV has been detected in multiple provinces across China (3–7). N-GoAstV infection can cause typical gout symptoms in goslings, with mortality rates exceeding 40% (8). Pathological examination reveals extensive urate deposition in the viscera, articular capsules, and cartilage, often accompanied by renal enlargement and related lesions (9). In recent years, the incidence of N-GoAstV infection in some waterfowl farms has exceeded 50% (10). The virus is mainly transmitted through both horizontal and vertical routes, posing an increasing threat to farmed populations and leading to considerable economic losses to the waterfowl industry (7, 11).

The complete genome of N-GoAstV comprises three open reading frames (ORF1a/b and ORF2) with partial sequence overlap (3). ORF1a and ORF1b primarily encode nonstructural proteins that participate in viral replication and transcription. The polyprotein encoded by ORF1a contains a 3C-like serine protease, which cleaves the polyprotein into several functional fragments (12). ORF1b encodes a highly conserved RNA-dependent RNA polymerase (RdRp), an essential enzyme that catalyzes virus RNA synthesis, including the production of a negative-sense RNA intermediate and the subsequent synthesis of positive-sense genomic and subgenomic RNAs (13). In contrast, ORF2 encodes the virus' capsid, is essential for virion assembly and host receptor binding, and also serves as an important antigenic determinant that elicits host immune responses (6, 13, 14). Notably, the C-terminal region of ORF2 contains a highly variable region, which serves as an important source of antigenic variation and host adaptation, as well as a hotspot for recombination events. Therefore, the ORF2 coding region is regarded as a suitable target for N-GoAstV genotyping and epidemiological studies, and is also a key focus for investigating genetic relationships and advancing vaccine development (15).

Current research on N-GoAstV has mainly concentrated on viral isolation, pathogenicity, and epidemiology. Although evolutionary analyses of N-GoAstV-1 and N-GoAstV-2 have been reported, a standardized genotyping framework remains lacking. In recent years, the Pairwise Sequence Comparison (PASC) method has been widely employed for the genotyping of various viruses, including duck circovirus, porcine circovirus (16–19). This method calculates pairwise sequence divergence (p-distance) and plots the frequency distribution of genetic distances to identify intra- and inter-genotype peaks. A threshold value is then selected at the valley between these peaks as the criterion for genotype demarcation. This approach reduces reliance on subjective interpretation of phylogenetic topology and provides a quantitative and reproducible framework for genotype and subtype classification. When distinct distributional peaks and valleys are present, candidate demarcation values can be identified from the valleys between peaks. Therefore, in this study, we applied the PASC approach to the ORF2 amino acid (aa) sequences of N-GoAstV, aiming to establish a quantitative and reproducible framework for genotype and subtype classification.

## 2 Material and methods

### 2.1 Collection of N-GoAstV genome sequences

To investigate the evolutionary characteristics and genotype classification of N-GoAstV, a total of 179 complete N-GoAstV genome sequences were accessed from the National Center for Biotechnology Information (NCBI) database (https://www.ncbi.nlm.nih.gov) as of 22 August 2025. The retrieved sequences were screened according to predefined inclusion criteria, including complete genome sequences, available host information, and the absence of obvious sequence gaps or incomplete genomic regions. No additional geographic or temporal sampling quotas were imposed. A total of 179 complete N-GoAstV genome sequences were retained for subsequent analyses. Detailed information for all included sequences, including GenBank accession numbers, strain names, host species, collection dates or years, is provided in Supplementary Table S1. Because the dataset was derived from publicly available GenBank records without predefined geographic or temporal sampling quotas, temporal and geographic distributions were interpreted as patterns of sequence availability in GenBank rather than population-level estimates of N-GoAstV subtype prevalence.

### 2.2 PASC-based genotype classification

To establish a quantitative and reproducible framework for N-GoAstV genotype and subtype classification, the Pairwise Sequence Comparison (PASC) method was applied to the amino acid (aa) sequences encoded by ORF2. Pairwise amino acid p-distances among the N-GoAstV sequences were calculated using MEGA X (Version 10.2.6) (Institute for Genomics and Evolutionary Medicine, Temple University, Philadelphia, USA) (20), with the p-distance defined as the proportion of amino acid differences per site between each pair of sequences. Ambiguous amino acid positions and alignment gaps were excluded from each pairwise comparison using the pairwise deletion option. The resulting pairwise p-distance values were exported from MEGA X for subsequent frequency distribution analysis. For visualization of the empirical p-distance distributions, frequency and relative-frequency distributions were generated in OriginPro 2022 (OriginLab Corporation, Northampton, MA, USA) using the software-generated automatic binning setting, which resulted in a bin width of 0.0016 for the present dataset. Candidate genetic-distance boundaries were identified from the observed distance-distribution patterns and interpreted in conjunction with the clustering patterns and branch topology of the corresponding NJ phylogenetic tree. Therefore, these values were treated as empirical demarcation boundaries rather than strict, topology-independent cutoffs. The resulting genotype and subtype assignments were subsequently evaluated using maximum-likelihood (ML) phylogenetic analysis. To further characterize the separation and overlap associated with the proposed groups, intra- and inter-group p-distance distributions were summarized according to the proposed genotype and subtype assignments.

### 2.3 Phylogenetic analysis

The ORF2 amino acid sequences were aligned using MEGA X (Version 10.2.6) with the MUSCLE algorithm under default parameters. The resulting alignment was used for subsequent phylogenetic analyses. To elucidate the evolutionary relationships of N-GoAstV, a maximum likelihood (ML) phylogenetic tree was built according to the ORF2 aa sequences. The optimal substitution model was determined using the Model Test function, and branch reliability was assessed with 1,000 bootstrap replications. A previously reported human astrovirus strain (NC_001943.1), which has been used in previous N-GoAstV phylogenetic studies, was included as an external reference for rooting the phylogenetic tree. Neighbor-joining (NJ) trees were constructed in MEGA X (Version 10.2.6) using the default settings without manual parameter adjustment. Separate NJ trees were generated for the complete N-GoAstV dataset and for the N-GoAstV-1 and N-GoAstV-2 subsets. The human astrovirus strain NC_001943.1 was included as the outgroup in the rooted NJ analyses. To evaluate whether the inferred N-GoAstV clustering pattern was sensitive to the choice of external outgroup, an additional unrooted ML tree was reconstructed using only the 179 N-GoAstV ORF2 amino acid sequences under the same substitution model and bootstrap settings as the rooted analysis.

### 2.4 Recombination analysis and sensitivity analysis

All 179 complete N-GoAstV genome sequences were screened for recombination using RDP4 with the RDP, GENECONV, BootScan, MaxChi, Chimera, SiScan, and 3Seq methods. Recombination signals supported by at least four independent methods were considered putative recombinant sequences. Because the primary objective of this study was to establish an ORF2-based classification framework, putative recombinant sequences were retained in the primary PASC and phylogenetic analyses. The predicted recombinant regions were further evaluated according to whether they overlapped with the ORF2 coding region. To assess whether ORF2-associated recombination affected the proposed classification, an additional sensitivity analysis was performed after excluding recombinant sequences with predicted recombination regions overlapping ORF2. The same phylogenetic parameters and rooting strategy were applied as in the primary ML analysis.

## 3 Descriptive results

In this study, the PASC (Pairwise Sequence Comparison) method was applied for the first time to systematically classify the ORF2 aa sequences of N-GoAstV. Previous studies have generally recognized two major genetic groups of N-GoAstV, corresponding to N-GoAstV-1 and N-GoAstV-2 based primarily on phylogenetic clustering. However, a unified and reproducible genotyping criterion has not yet been established (15). In the present study, the PASC-based classification was consistent with this previously recognized division, while providing quantitative information on genetic-distance distributions for further subdivision into five proposed subtypes. By calculating pairwise sequence p-distances and evaluating their frequency distributions in conjunction with the clustering patterns of neighbor-joining (NJ) phylogenetic trees, we supported the existence of the two major genotypes and further proposed a detailed subtyping framework.

As shown in Figure 1, the pairwise p-distance distribution of the ORF2 sequences exhibited several distinct components. A major transition was observed around a *p*-distance of 0.18, which, when evaluated together with the corresponding NJ topology, was associated with the separation of the two major phylogenetic groups, N-GoAstV-1 and N-GoAstV-2. Additional empirical boundaries were observed around 0.05 within N-GoAstV-1 and 0.033 within N-GoAstV-2. However, the separation between some intra- and inter-subtype distance distributions was not sharply defined. Therefore, the genetic-distance distributions were evaluated together with the clustering patterns and branch topology of the corresponding NJ phylogenetic tree. Within N-GoAstV-1, the proposed 0.05 boundary was supported by the separation between the N-GoAstV-1a/1b clusters and the lower intra-subtype distance variation observed within each group. The concordance between the major distance-distribution patterns and phylogenetic clustering supported the use of these values as empirical demarcation boundaries for genotype and subtype delineation. Based on this combined assessment, N-GoAstV-1 was further subdivided into N-GoAstV-1a, N-GoAstV-1b, and N-GoAstV-1c, whereas N-GoAstV-2 was subdivided into N-GoAstV-2a and N-GoAstV-2b. To further characterize the genetic-distance patterns underlying the proposed classification, pairwise p-distances were stratified according to genotype and subtype assignments. The corresponding intra- and inter-group distance statistics are summarized in Supplementary Table S3. The minor peaks located at approximately 0.19–0.22 in the overall distribution were primarily attributable to inter-subtype comparisons within N-GoAstV-1. Distances between N-GoAstV-1a and N-GoAstV-1b ranged from 0.1875 to 0.1946, whereas comparisons involving N-GoAstV-1c ranged from 0.2102 to 0.2164. In contrast, pairwise distances between N-GoAstV-1 and N-GoAstV-2 ranged from 0.5783 to 0.6065, revealing a broad genotype-level genetic-distance gap. At the subtype level within N-GoAstV-2, the distance distribution between N-GoAstV-2a and N-GoAstV-2b showed partial overlap, with inter-subtype distances ranging from 0.0071 to 0.0554. This overlap indicates that the boundary around 0.033 should not be interpreted as an absolute cutoff, but rather as an empirical demarcation reference supported by the corresponding phylogenetic clustering pattern. Taken together, the initial PASC/NJ analysis indicated an approximate genotype-level transition around 0.18, while the stratified pairwise-distance analysis further demonstrated strong genetic differentiation between the two major phylogenetic groups. Accordingly, the value around 0.18 was interpreted as an empirical demarcation reference within the combined PASC–phylogenetic framework rather than as a standalone pairwise-distance criterion.

**Figure 1:**
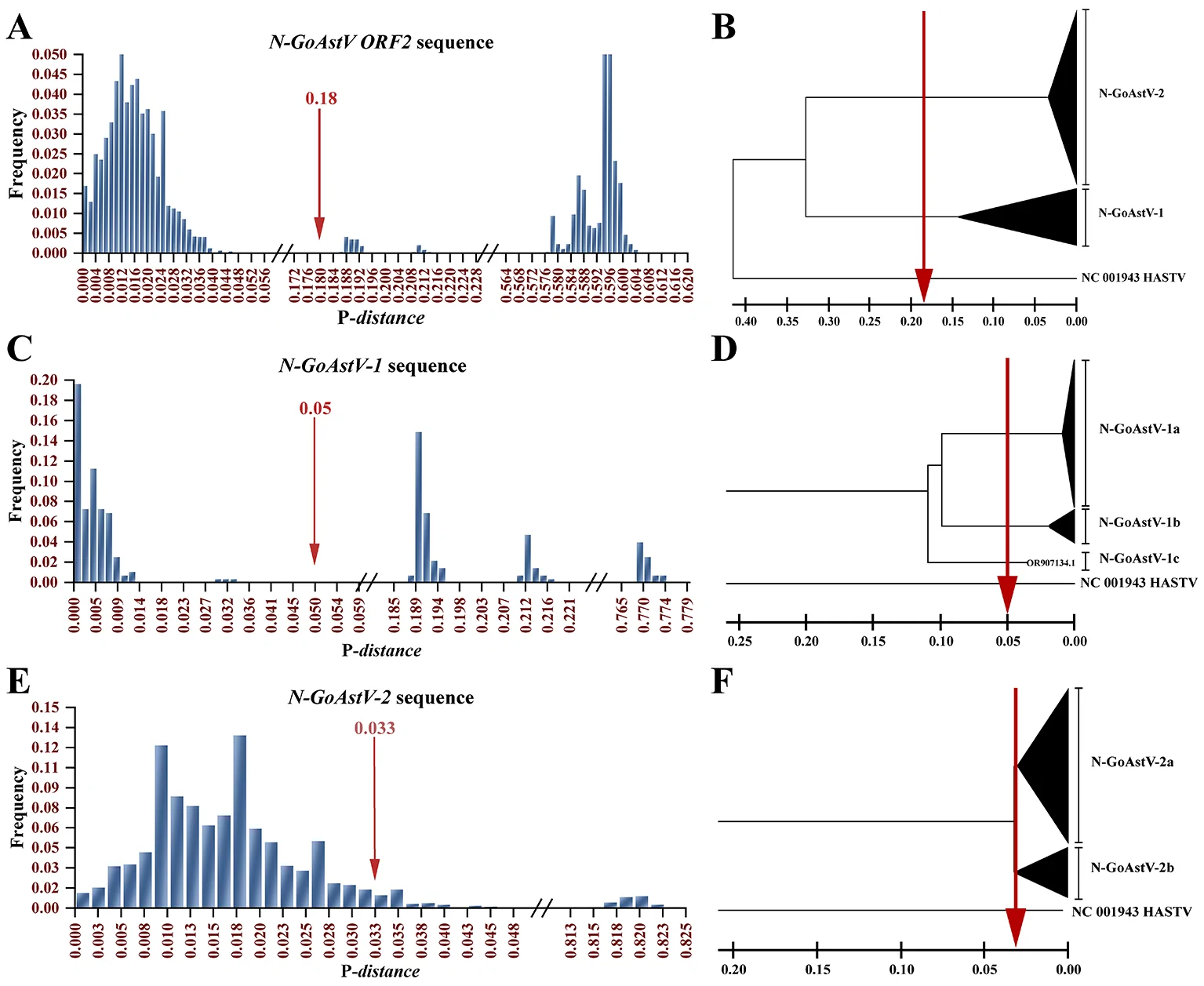
P-distance distribution and folded Neighbor-Joining (NJ) tree analysis of N-GoAstV ORF2 sequences. **(A)** Frequency distribution histogram of pairwise P-distances among 179 N-GoAstV ORF2 sequences. **(B)** Folded NJ tree constructed from the 179 N-GoAstV ORF2 sequences. **(C)** Frequency distribution histogram of pairwise P-distances among N-GoAstV-1 ORF2 sequences. **(D)** Folded NJ tree constructed from N-GoAstV-1 ORF2 sequences. **(E)** Frequency distribution histogram of pairwise P-distances among N-GoAstV-2 ORF2 sequences. **(F)** Folded NJ tree constructed from N-GoAstV-2 ORF2 sequences. All phylogenetic trees were rooted using a human astrovirus strain (NC_001943.1) as the outgroup.

Subsequently, a phylogenetic tree based on the ORF2 aa sequences was built using the maximum likelihood (ML) method with the optimal substitution model JTT+G (Figure 2). The N-GoAstV strains formed five proposed subtype-level phylogenetic groups corresponding to the proposed subtypes, and the overall clustering pattern was consistent with the proposed genotype and subtype assignments. The clustering pattern was largely consistent with the genotype and subtype assignments derived from the combined PASC and NJ analyses, providing additional phylogenetic support for the proposed classification. This approach not only corroborated the division into two major genotypes (N-GoAstV-1 and N-GoAstV-2) but also refined the proposed subtyping framework by integrating genetic-distance patterns with phylogenetic clustering, thereby providing a quantitative and reproducible basis for genotype and subtype demarcation. Although the placement of strain MN894548 differed slightly between the NJ and ML phylogenetic trees, the combined evidence supported its classification within the N-GoAstV-2a subtype. PASC analysis showed an average pairwise *p*-distance of 0.013 between MN894548 and N-GoAstV-2a reference strains, supporting its close genetic relationship with the N-GoAstV-2a group.

**Figure 2:**
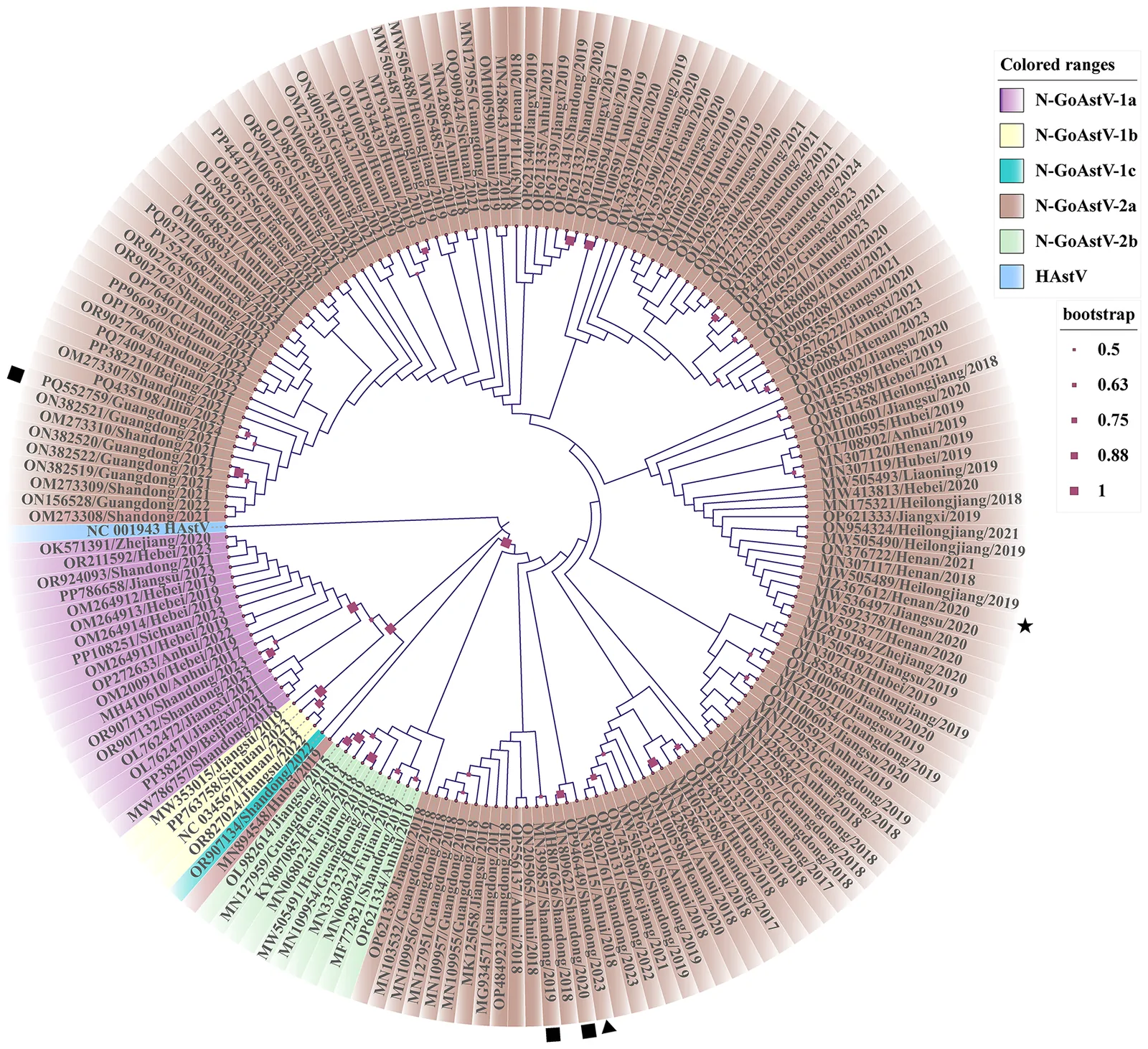
Maximum likelihood (ML) phylogenetic tree of N-GoAstV based on ORF2 sequences. The phylogenetic tree was built using MEGA X software via the best-fit substitution model JTT+G and analyzed by the maximum likelihood (ML) method. Branch support was assessed with 1,000 bootstrap replicates. Colored regions indicate the delineated genotypes and subtypes. A human astrovirus strain was used as the outgroup and is marked with a separate color. To distinguish N-GoAstV strains originating from different host species, duck-derived strains are indicated by black squares, chicken-derived strains by black triangles, and Muscovy duck–derived strains by black pentagrams. Strains not highlighted with color blocks represent goose-derived isolates.

To assess whether the observed subtype clustering was dependent on the rooting strategy, an additional unrooted ML tree was constructed using only the N-GoAstV ORF2 amino acid sequences (Supplementary Figure S1). The overall clustering pattern was consistent with that of the rooted ML tree, and the membership of the major proposed groups was retained. These results indicated that the principal N-GoAstV clustering pattern was not substantially affected by the placement of the human astrovirus outgroup.

Recombination screening identified 27 unique N-GoAstV sequences with putative recombination signals supported by at least four detection methods (Supplementary Table S2). MN894548 was not among these recombinant candidates. Of the identified recombination events, 11 overlapped the ORF2 coding region, whereas the remaining events occurred outside ORF2. Because the proposed classification was based on ORF2 amino acid variation, the potential impact of ORF2-associated recombination on genotype assignment was further evaluated by excluding sequences with recombination regions overlapping ORF2. In this sensitivity analysis, the major N-GoAstV-1/N-GoAstV-2 clustering pattern and multi-sequence subtype groupings were largely retained in the ORF2 ML phylogeny (Supplementary Figure S2), indicating that the observed classification framework was not substantially influenced by recombinant sequences. The N-GoAstV-1c lineage could not be independently evaluated because its sole representative strain (OR907134.1) was excluded due to an ORF2-overlapping recombination signal.

Among the sequences available in GenBank, N-GoAstV-2a was represented throughout 2016–2025 and accounted for the largest number of records in the present dataset, with most records collected during 2018–2021. N-GoAstV-1a was represented among sequences collected during 2017–2023, whereas N-GoAstV-2b was represented primarily among sequences collected during 2014–2019. N-GoAstV-2a was represented across the largest number of provinces in the available dataset. These patterns describe the composition of the available GenBank records and should not be interpreted as population-level prevalence or geographic incidence.

Astrovirus genomes contain three major coding regions, including ORF1a/1b and ORF2, which exhibit different evolutionary characteristics. Whole-genome sequences provide comprehensive evolutionary information; however, recombination events may complicate their application for routine classification. ORF1a and ORF1b encode nonstructural proteins involved in viral replication and polyprotein processing, and these regions have generally been less variable than ORF2 and less frequently used for astrovirus genotype demarcation. In contrast, ORF2 encodes the capsid protein, which is associated with host interaction, immune recognition, and antigenic variation (21). The higher variability and extensive availability of ORF2 sequences make this region suitable for distance-based classification approaches (8). Therefore, ORF2 amino acid sequences were selected as the basis for the proposed PASC framework.

Recent studies have revealed substantial genetic diversity and recombination among goose astroviruses (15). Genotype-associated variation in the ORF2-encoded capsid, including differences in antigenic epitopes, has also been reported (22), while recent experimental studies have further demonstrated genetic and pathogenic diversity among GAstV-2 strains (23). Similar to observations in other RNA viruses, structural variation of viral proteins may influence antigenicity and host interaction, although the biological consequences of N-GoAstV genetic diversification remain unclear (24). Future studies integrating comparative genomics, structural analyses, and experimental characterization will be required to determine the biological significance of the observed genetic diversification.

Several limitations should be considered when interpreting the proposed classification. First, the analyzed sequences were obtained from GenBank without systematic temporal or geographic sampling, and the observed subtype distribution therefore reflects database representation rather than population prevalence. Second, the proposed genetic-distance values represent empirical demarcation references rather than strict topology-independent cutoffs, particularly for N-GoAstV-2, in which intra- and inter-subtype distance distributions partially overlapped. Third, N-GoAstV-1c is currently represented by a single sequence that showed an ORF2-overlapping recombination signal; therefore, its designation should be regarded as provisional until additional non-recombinant sequences become available.

In conclusion, we identified five proposed subtype-level groups (N-GoAstV-1a–c and N-GoAstV-2a–b), with N-GoAstV-1c remaining provisional, and established a refined framework for describing N-GoAstV genetic diversity. The PASC-based approach provided a quantitative and reproducible framework for genotype and subtype delineation based on empirical genetic-distance patterns and complementary phylogenetic support, thereby providing a quantitative framework that complements phylogenetic classification by incorporating genetic-distance information. The observed subtype distribution reflects the available GenBank sequence dataset and should not be interpreted as a direct estimate of population-level prevalence. This work provides a quantitative framework for describing the genetic diversity of N-GoAstV and may serve as a reference for future molecular epidemiological investigations, vaccine development, and disease prevention and control.

## Data Availability

The original contributions presented in the study are included in the article/Supplementary material, further inquiries can be directed to the corresponding author.
