# Supplementary material for: Novel PASC-based genotyping of goose astrovirus using ORF2 amino acid sequences
Source: Front Vet Sci. 2026 Oct 27;13:1732594. doi: 10.3389/fvets.2026.1732594 (PMC13747832; doi:10.3389/fvets.2026.1732594)
Supplement: Supplementary file 1 [file Data_Sheet_1.docx]

Supplementary Material

PASC-Based Genotyping of Novel Goose Astrovirus Using ORF2 Amino Acid Sequences

Zhibin Zhang^1^, Haoyang Wang^1^, Xin Xu^1^, Dandan Li^1^, Jun Ji^1^, *, Lunguang Yao^1^, Yingzuo Bi^2^, Qingmei Xie^2^

**Correspondence:** Corresponding Author: [jijun020@126.com](mailto:jijun020@126.com)

**Supplementary Table S1.** Summary information of the 179 N-GoAstV genome sequences included in this study.

| **Accession** | **Genotype** | **Strain Name** | **Host Species** | **Collection Date** | **Country** | **Province/Region** |
| --- | --- | --- | --- | --- | --- | --- |
| OR827024 | N-GoAstV-1b | GAstV-JSXZ | goose | 6-Sep-22 | China | Jiangsu |
| OP946449 | N-GoAstV-2a | GAstV-SDHZ | chicken | 29-Oct-23 | China | Shandong |
| OP621341 | N-GoAstV-2a | AAstV/Goose/CHN/2019/SD03 | goose | 2019/10/9 | China | Shandong |
| OP621340 | N-GoAstV-2a | AAstV/Goose/CHN/2019/JX03 | goose | 2019/12/2 | China | Jiangxi |
| OP621339 | N-GoAstV-2a | AAstV/Goose/CHN/2019/JX02 | goose | 2019/12/2 | China | Jiangxi |
| OP621338 | N-GoAstV-2a | AAstV/Goose/CHN/2018/JX01 | goose | 2018/3/29 | China | Jiangxi |
| OP621337 | N-GoAstV-2a | AAstV/Goose/CHN/2018/JS01 | goose | 2018/2/6 | China | Jiangsu |
| OP621335 | N-GoAstV-2a | AAstV/Goose/CHN/2021/AH02 | goose | 2021/5/8 | China | Anhui |
| OP621334 | N-GoAstV-2b | AAstV/Goose/CHN/2019/AH01 | goose | 2019/11/15 | China | Anhui |
| OP621333 | N-GoAstV-2a | AAstV/Goose/CHN/2019/JX-Embryo | goose | 2019/12/16 | China | Jiangxi |
| OP621332 | N-GoAstV-2a | AAstV/Goose/CHN/2020/SD04 | goose | 2020/11/18 | China | Shandong |
| OP621331 | N-GoAstV-2a | AAstV/Goose/CHN/2019/SD02 | goose | 2019/5/3 | China | Shandong |
| OP621330 | N-GoAstV-2a | AAstV/Goose/CHN/2021/JX04 | goose | 2021/3/16 | China | Jiangxi |
| MW786757 | N-GoAstV-1a | SCCD | goose | Sep-18 | China | Shandong |
| OM273310 | N-GoAstV-2a | G576 | goose | 21-Aug-21 | China | Shandong |
| OM273309 | N-GoAstV-2a | G548 | goose | 16-Jul-21 | China | Shandong |
| OM273308 | N-GoAstV-2a | G538 | goose | 30-Jun-21 | China | Shandong |
| OM273307 | N-GoAstV-2a | G529 | goose | 7-May-21 | China | Shandong |
| OM273306 | N-GoAstV-2a | G520 | goose | 26-Apr-21 | China | Shandong |
| OM273305 | N-GoAstV-2a | G519 | goose | 15-Apr-21 | China | Shandong |
| OM273304 | N-GoAstV-2a | G518 | goose | 10-Apr-21 | China | Shandong |
| OM273303 | N-GoAstV-2a | G516 | goose | 28-Mar-21 | China | Shandong |
| OM273302 | N-GoAstV-2a | G512 | goose | 12-Mar-21 | China | Shandong |
| MN809622 | N-GoAstV-2a | AstV/SDTA | duck | 17-Jun-20 | China | Shandong |
| MH052598 | N-GoAstV-2a | AstV/SDPY/Goose/1116/17 | goose | 16-Nov-17 | China | Shandong |
| PQ740944 | N-GoAstV-2a | CH/HBXT/2022 | goose | 2022 | China | Henan |
| PQ308726 | N-GoAstV-2a | GAstV_GDFS_Goose_2024 | goose | 2024 | China | Guangdong |
| PP444710 | N-GoAstV-2a | GAstV GDCS Goose 2023 | goose | 2023 | China | Guangdong |
| PP444709 | N-GoAstV-2a | GAstV_GXNN_Goose_2023 | goose | 2023 | China | Guangxi |
| PP108251 | N-GoAstV-1a | AAstV/Goose/CHN/2023/C1 | goose | 15-Aug-23 | China | Sichuan |
| OR965521 | N-GoAstV-2a | AAstV/Goose/CHN/2023/MG-23 | goose | Jul-23 | China | Anhui |
| OR958817 | N-GoAstV-2a | AAstV/Goose/CHN/2023/HR2306/1 | goose | Jun-23 | China | Anhui |
| OR907134 | N-GoAstV-1c | G2332 | goose | 26-Oct-22 | China | Shandong |
| OR907132 | N-GoAstV-1a | A1082 | goose | 7-Mar-23 | China | Shandong |
| OR907131 | N-GoAstV-1a | C1330 | goose | 21-Mar-23 | China | Shandong |
| ON400505 | N-GoAstV-2a | GAstV-GD2104 | goose | 1-Dec-21 | China | Guangdong |
| ON382522 | N-GoAstV-2a | GAstV-GD2105 | goose | 15-Jan-22 | China | Guangdong |
| ON382521 | N-GoAstV-2a | GAstV-GD2103 | goose | 16-Nov-21 | China | Guangdong |
| ON382520 | N-GoAstV-2a | GAstV-GD2102 | goose | 16-Nov-21 | China | Guangdong |
| ON382519 | N-GoAstV-2a | GAstV-GD2101 | goose | 26-Oct-21 | China | Guangdong |
| OP764611 | N-GoAstV-2a | AH-16 | goose | 2021 | China | Anhui |
| OP776629 | N-GoAstV-2a | GDZJ-21-01 | goose | 2021 | China | Guangdong |
| OP272633 | N-GoAstV-1a | AH33-3 | goose | 2022 | China | Anhui |
| OP484924 | N-GoAstV-2a | AAstV/Goose/CHN/2018/HB01 | goose | 2018/4/11 | China | Hubei |
| OP484923 | N-GoAstV-2a | AAstV/Goose/CHN/2018/GD01 | goose | 2018/3/9 | China | Guangdong |
| ON954324 | N-GoAstV-2a | GAsTV/2021/CHN | goose | 2021 | China | Heilongjiang |
| ON205965 | N-GoAstV-2a | AAstv/CHN/Goose/2018 | goose | May-18 | China | Anhui |
| OM811458 | N-GoAstV-2a | Astv/Goose/Heilongjiang/ZYL01/2018 | goose | 31-Dec-18 | China | Heilongjiang |
| OM066895 | N-GoAstV-2a | AAstV/Goose/CHN/2021/ZY02 | goose | Apr-21 | China | Anhui |
| OM066894 | N-GoAstV-2a | AAstV/Goose/CHN/2021/HR2110/1 | goose | Oct-21 | China | Anhui |
| OM066893 | N-GoAstV-2a | AAstV/Goose/CHN/2021/HR2105/2 | goose | May-21 | China | Anhui |
| OM066892 | N-GoAstV-2a | AAstV/Goose/CHN/2021/HR2105/1 | goose | May-21 | China | Anhui |
| OM100603 | N-GoAstV-2a | AstV/JS04/Goose/0919/20 | goose | 19-Sep-20 | China | Jiangsu |
| OM100602 | N-GoAstV-2a | AstV/JS03/Goose/0702/20 | goose | 2-Jul-20 | China | Jiangsu |
| OM100601 | N-GoAstV-2a | AstV/JS02/Goose/0518/20 | goose | 18-May-20 | China | Jiangsu |
| OM100600 | N-GoAstV-2a | AstV/JS01/Goose/0518/20 | goose | 18-May-20 | China | Jiangsu |
| OM100599 | N-GoAstV-2a | AstV/HN05/Goose/0514/20 | goose | 14-May-20 | China | Henan |
| OM100598 | N-GoAstV-2a | AstV/HN04/Goose/0514/20 | goose | 14-May-20 | China | Henan |
| OM100597 | N-GoAstV-2a | AstV/HB06/Goose/1226/19 | goose | 26-Dec-19 | China | Hubei |
| OM100596 | N-GoAstV-2a | AstV/HB05/Goose/0812/19 | goose | 12-Aug-19 | China | Hubei |
| OM100595 | N-GoAstV-2a | AstV/HB04/Goose/0811/19 | goose | 12-Aug-19 | China | Hubei |
| OM100594 | N-GoAstV-2a | AstV/HB03/Goose/0810/19 | goose | 11-Aug-19 | China | Hubei |
| OM100593 | N-GoAstV-2a | AstV/AN06/Goose/0714/19 | goose | 14-Jul-19 | China | Anhui |
| OM100592 | N-GoAstV-2a | AstV/AH05/Goose/0711/19 | goose | 11-Jul-19 | China | Anhui |
| OM100591 | N-GoAstV-2a | AstV/AH04/Goose/0614/19 | goose | 14-Jun-19 | China | Anhui |
| OM100590 | N-GoAstV-2a | AstV/AH03/Goose/0422/19 | goose | 22-Apr-19 | China | Anhui |
| OP020131 | N-GoAstV-2a | AAstV/Goose/CHN/2022/SD05 | goose | 2022/1/16 | China | Shandong |
| MW505493 | N-GoAstV-2a | AstV/Goose/2019/LNAS | goose | 21-Aug-19 | China | Liaoning |
| MW505492 | N-GoAstV-2a | AstV/Goose/2019/JSXZ | goose | 20-Jun-19 | China | Jiangsu |
| MW505491 | N-GoAstV-2b | AstV/Goose/2019/HLJYC | goose | 6-Jun-19 | China | Heilongjiang |
| MW505490 | N-GoAstV-2a | AstV/Goose/2019/HLJJX | goose | 21-Jun-19 | China | Heilongjiang |
| MW505489 | N-GoAstV-2a | AstV/Goose/2019/HLJSYS | goose | 21-Jun-19 | China | Heilongjiang |
| MW505488 | N-GoAstV-2a | AstV/Goose/2019/HLJJMS | goose | 16-Jul-19 | China | Heilongjiang |
| MW505487 | N-GoAstV-2a | AstV/Goose/2019/HLJHRB | goose | 11-Jun-19 | China | Heilongjiang |
| MW505486 | N-GoAstV-2a | AstV/Goose/2019/SDWF | goose | 5-Jul-19 | China | Shandong |
| MW505485 | N-GoAstV-2a | AstV/Goose/2019/JLSL | goose | 5-Aug-19 | China | Jilin |
| MZ648231 | N-GoAstV-2a | AAstV/Goose/CHN/2021/ZM | goose | 2021 | China | Anhui |
| MT708902 | N-GoAstV-2a | AAstV/Goose/CHN/2019/DY-19 | goose | Sep-19 | China | Anhui |
| MN109957 | N-GoAstV-2a | Astv/Goose/Guangdong/1810MFC/2018 | goose | 11-Oct-18 | China | Guangdong |
| MN109956 | N-GoAstV-2a | Astv/Goose/Guangdong/1808CCF/2018 | goose | 25-Aug-18 | China | Guangdong |
| MN109955 | N-GoAstV-2a | Astv/Goose/Guangdong/1803LZM/2018 | goose | 9-Mar-18 | China | Guangdong |
| MN109954 | N-GoAstV-2b | Astv/Goose/Guangdong/1807LWG/2018 | goose | 1-Jul-18 | China | Guangdong |
| MN127959 | N-GoAstV-2b | Astv/Goose/Guangdong/1812LMG/2018 | goose | 18-Dec-18 | China | Guangdong |
| OR906207 | N-GoAstV-2a | HNxy2106 | goose | 2021 | China | Henan |
| MN127958 | N-GoAstV-2a | Astv/Goose/Guangdong/1811LHC/2018 | goose | 30-Nov-18 | China | Guangdong |
| MN127957 | N-GoAstV-2a | Astv/Goose/Guangdong/1811DWM/2018 | goose | 29-Nov-18 | China | Guangdong |
| MN127956 | N-GoAstV-2a | Astv/Goose/Guangdong/1811CFC/2018 | goose | 25-Nov-18 | China | Guangdong |
| MN127955 | N-GoAstV-2a | Astv/Goose/Guangdong/1901SM/2019 | goose | 16-Jan-19 | China | Guangdong |
| MN127954 | N-GoAstV-2a | Astv/Goose/Guangdong/1901TBF/2019 | goose | 16-Jan-19 | China | Guangdong |
| MN127953 | N-GoAstV-2a | Astv/Goose/Guangdong/1901LNC/2019 | goose | 3-Jan-19 | China | Guangdong |
| MN127952 | N-GoAstV-2a | Astv/Goose/Guangdong/1812GXG/2018 | goose | 19-Dec-18 | China | Guangdong |
| MN127951 | N-GoAstV-2a | Astv/Goose/Guangdong/1811TS/2018 | goose | 20-Nov-18 | China | Guangdong |
| MN103532 | N-GoAstV-2a | Astv/Goose/Guangdong/1807LZQ/2018 | goose | 2018/7/1 | China | Guangdong |
| MN307120 | N-GoAstV-2a | AstV/HN03/Goose/0402/19 | goose | 2-Apr-19 | China | Henan |
| MN307119 | N-GoAstV-2a | AstV/HB02/Goose/0310/19 | goose | 10-Mar-19 | China | Hubei |
| MN307118 | N-GoAstV-2a | AstV/HB01/Goose/0123/19 | goose | 3-Jan-19 | China | Hubei |
| MN307117 | N-GoAstV-2a | AstV/HN02/Goose/1119/18 | goose | 19-Nov-18 | China | Henan |
| MN307116 | N-GoAstV-2a | AstV/AH02/Goose/0715/18 | goose | 15-Jul-18 | China | Anhui |
| MN307115 | N-GoAstV-2a | AstV/AH01/Goose/0512/18 | goose | 12-May-18 | China | Anhui |
| MN307114 | N-GoAstV-2a | AstV/HN01/Goose/0103/18 | goose | 3-Jan-18 | China | Henan |
| MN894548 | N-GoAstV-2a | AstV/HBXG/Goose/2019 | goose | 16-Jun-19 | China | Hubei |
| MN428645 | N-GoAstV-2a | AHAU5 | goose | 2018 | China | Anhui |
| MN428644 | N-GoAstV-2a | AHAU4 | goose | 2018 | China | Anhui |
| MN428643 | N-GoAstV-2a | AHAU3 | goose | 2018 | China | Anhui |
| MN428642 | N-GoAstV-2a | AHAU2 | goose | 2018 | China | Anhui |
| MN428641 | N-GoAstV-2a | AHAU1 | goose | 2018 | China | Anhui |
| MN175321 | N-GoAstV-2a | AstV/Goose/2018/HLJ01 | goose | 9-Jul-18 | China | Heilongjiang |
| MN068024 | N-GoAstV-2b | GTF-07 | goose | Apr-18 | China | Fujian |
| MN068023 | N-GoAstV-2b | GTF-04 | goose | Apr-18 | China | Fujian |
| MN337323 | N-GoAstV-2b | XX | goose | 10-Jul-18 | China | Henan |
| MK125058 | N-GoAstV-2a | JSHA | goose | 2016 | China | Jiangsu |
| MH807626 | N-GoAstV-2a | AstV/Goose/CXZ/18 | goose | 8-Jan-18 | China | Shandong |
| KY807085 | N-GoAstV-2b | HN1G | goose | 20-May-14 | China | Henan |
| MH410610 | N-GoAstV-1a | AHDY | goose | Aug-17 | China | Anhui |
| NC_034567 | N-GoAstV-1b | FLX | goose | 4-Jun-14 | China | Hunan |
| MG934571 | N-GoAstV-2a | GD | goose | 1-Nov-17 | China | Guangdong |
| MF772821 | N-GoAstV-2b | AAstV/Goose/CHN/2017/SD01 | goose | Apr-17 | China | Shandong |
| MT934439 | N-GoAstV-2a | HNU-CSZ2-2019 | goose | May-19 | China | Hunan |
| MT934438 | N-GoAstV-2a | HNU-LYG3-2019 | goose | May-19 | China | Hunan |
| MT934437 | N-GoAstV-2a | HNU-LYG2-2019 | goose | May-19 | China | Hunan |
| PV524668 | N-GoAstV-2a | JXNC1 | goose | 15-Mar-25 | China | Jiangxi |
| PQ552759 | N-GoAstV-2a | GD2406 | duck | 2024 | China | Guangdong |
| PQ435198 | N-GoAstV-2a | JL2309 | goose | 2023 | China | Jilin |
| PP966939 | N-GoAstV-2a | GZ2301 | goose | 2023 | China | Guizhou |
| PP763758 | N-GoAstV-1b | AAstV/Goose/CHN/2023/C102 | goose | Jun-23 | China | Sichuan |
| OM455389 | N-GoAstV-2a | HeB-BD4-2019 | goose | 16-Sep-19 | China | Hebei |
| OM455388 | N-GoAstV-2a | HeB-BD1-2021 | goose | 24-Jan-21 | China | Hebei |
| OM264914 | N-GoAstV-1a | HeB-SJZ-2019 | goose | 4-Jan-19 | China | Hebei |
| OM264913 | N-GoAstV-1a | HeB-BD-1-2019 | goose | 6-Jul-19 | China | Hebei |
| OM264912 | N-GoAstV-1a | HeB-BD-2-2019 | goose | 15-Jul-19 | China | Hebei |
| OM264911 | N-GoAstV-1a | HeB-CZ-2019 | goose | 19-Jul-19 | China | Hebei |
| OM200916 | N-GoAstV-1a | HeB-BD-3-2019 | goose | 20-Jul-19 | China | Hebei |
| OL536330 | N-GoAstV-2a | HeB-BD1-2020 | goose | 21-Oct-20 | China | Hebei |
| OL762472 | N-GoAstV-1a | JXYC | goose | 7-Dec-21 | China | Jiangxi |
| OL762471 | N-GoAstV-1a | JXGZ | goose | 7-Dec-21 | China | Jiangxi |
| PP786658 | N-GoAstV-1a | JY202323 | goose | 3-Feb-23 | China | Jiangsu |
| PQ037214 | N-GoAstV-2a | SDWF | goose | 2024 | China | Shandong |
| OQ909424 | N-GoAstV-2a | SCG3 | goose | 9-May-21 | China | Sichuan |
| OP621336 | N-GoAstV-2a | AAstV/Goose/CHN/2018/HB02 | goose | 2018/4/22 | China | Hubei |
| ON376722 | N-GoAstV-2a | HNZZ-4 | goose | Dec-21 | China | Henan |
| MW353015 | N-GoAstV-1b | TZ03 | goose | 7-Aug-19 | China | Jiangsu |
| MZ367612 | N-GoAstV-2a | HNNY0620 | Muscovy-duck | 20-Jun-20 | China | Henan |
| MW592379 | N-GoAstV-2a | HNXX-6/China/2020 | goose | 2020 | China | Henan |
| MW592378 | N-GoAstV-2a | HNSQ-6/China/2020 | goose | 2020 | China | Henan |
| MW592377 | N-GoAstV-2a | HNKF-1/China/2020 | goose | 2020 | China | Henan |
| MW345727 | N-GoAstV-2a | SDPD | goose | Jan-19 | China | Shandong |
| MN399857 | N-GoAstV-2a | SDXT | duck | 15-Mar-19 | China | Shandong |
| PV600843 | N-GoAstV-2a | HNJZ-2023 | goose | 2023 | China | Henan |
| PP382210 | N-GoAstV-2a | J2/2021 | goose | 14-Oct-21 | China | Beijing |
| PP382209 | N-GoAstV-1a | J3/2021 | goose | 14-Oct-21 | China | Beijing |
| OR906208 | N-GoAstV-2a | HNxy2111 | goose | 2021 | China | Henan |
| OR924093 | N-GoAstV-1a | L1002 | goose | 10-May-21 | China | Shandong |
| OR902765 | N-GoAstV-2a | U50 | goose | 19-Jun-23 | China | Shandong |
| OR902764 | N-GoAstV-2a | U52 | goose | 21-Jun-23 | China | Shandong |
| OR902763 | N-GoAstV-2a | U56 | goose | 25-Jun-23 | China | Shandong |
| OR902762 | N-GoAstV-2a | U61 | goose | 10-Jul-23 | China | Shandong |
| OR902761 | N-GoAstV-2a | Q2001 | goose | 21-Oct-23 | China | Shandong |
| OR211592 | N-GoAstV-1a | GAstV-HBLY | goose | 2023 | China | Hebei |
| OP179660 | N-GoAstV-2a | GoAstv-C2 | goose | 25-Jun-22 | China | Sichuan |
| OP556137 | N-GoAstV-2a | AHQJ18 | goose | Jul-18 | China | Anhui |
| ON156528 | N-GoAstV-2a | GDZJ37 | goose | 10-Jan-22 | China | Guangdong |
| ON745304 | N-GoAstV-2a | ZJCX | goose | 15-Jun-21 | China | Zhejiang |
| OM937013 | N-GoAstV-2a | SD | goose | May-17 | China | Shandong |
| OL853843 | N-GoAstV-2a | HLJ | goose | 2019 | China | Heilongjiang |
| OL982615 | N-GoAstV-2a | JSZ29 | goose | 11-Oct-20 | China | Jiangsu |
| OL982614 | N-GoAstV-2b | JSCZ15 | goose | 11-Nov-15 | China | Jiangsu |
| OL982613 | N-GoAstV-2a | XSSH | goose | 10-Jun-20 | China | Zhejiang |
| OL963558 | N-GoAstV-2a | JSW10 | goose | 24-Sep-20 | China | Jiangsu |
| OL963557 | N-GoAstV-2a | JSW1 | goose | 3-Sep-20 | China | Jiangsu |
| OL963556 | N-GoAstV-2a | JSSQ | goose | 11-Jul-20 | China | Jiangsu |
| OL963555 | N-GoAstV-2a | JSCZ | goose | 15-Jun-20 | China | Jiangsu |
| MZ819184 | N-GoAstV-2a | ZJLD | goose | Jun-20 | China | Zhejiang |
| MW536497 | N-GoAstV-2a | YC20 | goose | 10-Aug-20 | China | Jiangsu |
| OK148600 | N-GoAstV-2a | SQ | goose | 2-Oct-20 | China | Jiangsu |
| MZ540211 | N-GoAstV-2a | JS2019/China | goose | 2019 | China | Jiangsu |
| OK571391 | N-GoAstV-1a | ZJC14 | goose | 10-Jun-20 | China | Zhejiang |
| OK571390 | N-GoAstV-2a | JRMS | goose | 3-Dec-20 | China | Zhejiang |
| MZ576222 | N-GoAstV-2a | JX01/China/2021 | goose | 2021/5/20 | China | Jiangxi |
| MW413813 | N-GoAstV-2a | XT1 | goose | 5-May-20 | China | Hebei |

**Supplementary Table S2.** Putative recombinant N-GoAstV sequences identified using RDP4.

| **No.** | **Recombinant**  **Sequence** | **Breakpoint Positions** | **Minor parent** | **Major parent** | **ORF2 overlap** |
| --- | --- | --- | --- | --- | --- |
| 1 | OR907134.1 | 4664–5926 | OR827024.1 | OK571391.1 | Yes |
| 2 | ON382521.1 | 7289–7328 | OL762471.1 | OM273309.1 | No |
| 3 | PV524668.1 | 2386–7196 | OR902763.1 | MZ576222.1 | Yes |
| 4 | MH052598.1 | 7268–171 | OR902765.1 | OP621336.1 | No |
| 5 | OL853843.1 | 1–46 | OM066894.1 | MN127959.1 | No |
| 6 | MW505492.1 | 7268–7330 | MN109957.1 | OP776629.1 | No |
| 7 | OL982614.1 | 7256–2445 | MH807626.1 | MW592378.1 | No |
| 8 | MW505485.1 | 7302–363 | OR906207.1 | OP621334.1 | No |
| 9 | OK148600.1 | 379–2654 | MN103532.1 | OL963555.1 | No |
| 10 | PP382210.1 | 1192–1592 | MW345727.1 | ON382522.1 | No |
| 11 | OM937013.1 | 453–2500 | MW592378.1 | MN109955.1 | No |
| 12 | MN127953.1 | 152–1620 | MN103532.1 | MN127952.1 | No |
| 13 | OP621335.1 | 1557–5268 | OM273303.1 | MN894548.1 | Yes |
| 14 | MN307116.1 | 3008–5943 | MN307119.1 | OP946449.1 | Yes |
| 15 | OL982615.1 | 2752–5344 | MW592379.1 | OL982613.1 | Yes |
| 16 | MN307120.1 | 2474–6782 | OQ909424.1 | MN109955.1 | Yes |
| 17 | MN307114.1 | 5617–7186 | MW505485.1 | OL982614.1 | Yes |
| 18 | OP621332.1 | 5260–6276 | OP621334.1 | OM066895.1 | Yes |
| 19 | MW353015.1 | 4888–7214 | NC_034567.1 | OM264912.1 | Yes |
| 20 | OR827024.1 | 7214–3461 | MH410610.1 | PP108251.1 | No |
| 21 | PP763758.1 | 4862–7212 | NC_034567.1 | OR907132.1 | Yes |
| 22 | NC_034567.1 | 2924–3461 | OR907134.1 | OM200916.1 | No |
| 23 | MN809622.1 | 150–1652 | PP966939.1 | MK125058.1 | No |
| 24 | MN127959.1 | 3550–7068 | KY807085.1 | MN809622.1 | Yes |
| 25 | OP621334.1 | 856–1698 | OM273303.1 | MK125058.1 | No |
| 26 | OM100600.1 | 1128–1283 | ON156528.1 | MN399857.1 | No |
| 27 | MN399857.1 | 405–2352 | OM066895.1 | OP621338.1 | No |

**Supplementary Table S3.** Intra- and inter-group amino acid p-distance distributions supporting PASC-based genotype and subtype boundaries.

| **Comparison** | **Number of comparisons** | **Mean**  **p-distance** | **Median** | **Minimum** | **Maximum** | **95th percentile** |
| --- | --- | --- | --- | --- | --- | --- |
| Within N-GoAstV-1a | 153 | 0.037 | 0.0043 | 0 | 0.0128 | 0.0085 |
| Within N-GoAstV-1b | 6 | 0.0184 | 0.0184 | 0.0042 | 0.0325 | 0.0322 |
| Within N-GoAstV-2a | 10585 | 0.0135 | 0.0128 | 0 | 0.0426 | 0.0241 |
| Within N-GoAstV-2b | 45 | 0.0179 | 0.0170 | 0 | 0.0327 | 0.0284 |
| N-GoAstV-1 vs N-GoAstV-2 | 3431 | 0.596 | 0.595 | 0.5827 | 0.6065 | — |
| N-GoAstV-1a vs N-GoAstV-1b | 72 | 0.1907 | 0.1903 | 0.1875 | 0.1946 | — |
| N-GoAstV-1a vs N-GoAstV-1c | 18 | 0.2121 | 0.2116 | 0.2102 | 0.2159 | — |
| N-GoAstV-1b vs N-GoAstV-1c | 4 | 0.2136 | 0.2129 | 0.2122 | 0.2164 | — |
| N-GoAstV-2a vs N-GoAstV-2b | 1460 | 0.0257 | 0.0256 | 0.0071 | 0.0554 | — |

**Supplementary Figure S1:** Unrooted maximum-likelihood phylogenetic tree based on ORF2 amino acid sequences of N-GoAstV strains. The tree was reconstructed using the same ORF2 amino acid alignment and phylogenetic parameters as the rooted ML tree, except that no external outgroup was included. Branch support values were calculated using 1,000 bootstrap replicates. Different colors indicate the proposed genotype and subtype assignments. The clustering pattern was consistent with the rooted ML phylogenetic tree, indicating that the proposed N-GoAstV classification was not substantially affected by the selection of the human astrovirus outgroup.

**Supplementary Figure S2**. Maximum-likelihood phylogenetic tree of N-GoAstV ORF2 amino acid sequences after exclusion of sequences with ORF2-overlapping recombination signals. Maximum-likelihood phylogenetic tree of N-GoAstV ORF2 amino acid sequences after exclusion of sequences with ORF2-overlapping recombination signals.
